# A Sporting and Peronist Youth in the Making: The High School Students Union (UES) in Argentina (1952–1955)

**DOI:** 10.3389/fspor.2020.568282

**Published:** 2020-11-12

**Authors:** Lucie Hémeury

**Affiliations:** Centre de Recherche et de Documentation des Amériques, Institut des Hautes Études d'Amérique latine, Université Sorbonne Nouvelle, Paris, France

**Keywords:** Argentina, Peronism, youth, sports, UES

## Abstract

This article examines the history of the High School Secondary Students (*Unión de Estudiantes Secundarios* or UES), a sporting and cultural youth association created by the Peronist government in 1953. From its inception, this institution has been compared by sectors of the opposition to Fascist and Nazi youth organizations and accused of politically enlisting and morally corrupting Argentine youth. Based on institutional archives, UES publications and Perón's speeches, this study offers a contribution to the historiographical debates on the history of youth, sports and Peronism. It shows how the creation of the UES relates to the social, educational and sports policies of this regime. The article analyses the objectives pursued by the government, but also the limits and contradictions of this attempt to mobilize young people through sports activities.

## Introduction

Between 1946 and 1955, the government led by Juan Domingo Perón implemented a wide-ranging sports policy. Perón, a former military fencing champion and an accomplished sportsman, was a fervent advocate of the practice of sport and sincerely convinced of its pedagogical and educational virtues. Since the 1920 and 1930's, sport gradually became an important and valuable activity in Europe and the United States, with many benefits and which, as such, should be encouraged by the public authorities (Lanfranchi, 2000; Darbon, 2008). In Argentina, the radical and then conservative governments of the 1920 and 1930's also undertook the promotion of the sports practice among children and adolescents. The Peronist government multiplied and systematized these first disparate and isolated experiences, giving them a national scope and articulating them to their health, social and cultural policy programs (Hémeury, 2018, p. 43–101).

The proclaimed objectives of the Peronist sports policy—as exposed in the 1952–1957 second 5-years plan—responded to Perón's wish to forge “a nation of sportsmen^1^.” The government undertook to provide financial support both for high-performance sport and for the popular and mass practice of physical activities. As sport was perceived as a fundamental element of education which contributed to the physical and moral development of young people, children and teenagers were primary targets of the measures adopted during this decade.

The regime created specific organizations for them: the first one was the Eva Perón Foundation (*Fundación Eva Perón* or FEP), a welfare institution founded as early as 1948 by the Argentinean First Lady, Eva Perón (Plotkin, 2003; Barry et al., 2008). Soon this social welfare organization, aimed at the most disadvantaged sectors of society, especially women and children, became a major protagonist in national sports policy, organizing the Evita Children's Tournaments (*Campeonatos Infantiles Evita* or *Torneos Evita*) with increasing success. This annual sports competition for children, open to girls since 1952, shows the decisive role played by sport in the Peronist programs for the youngest and the importance attached to the care of children by the government. Indeed, for the Peronist leaders, children were the “only privileged of the nation^2^.”

Compared to the FEP, the High School Students Union (*Unión de Estudiantes Secundarios* or UES), the second Peronist sports institution created for young people, was a latecomer organization since it was founded in 1953 by the then Minister of Education, Armando Méndez San Martín. It indicates the priority given to young children over adolescents in the early years of the regime. Actually, the distinction between the two categories was not yet clear: as Manzano pointed out, the Peronist period, and especially the last years of Perón's second term of office, represents a transitional phase that contributed to the emergence of the concept of “youth” as a generational, social and cultural category distinct from childhood and adulthood. Adolescents increasingly tended to be considered by public authorities as a separate category raising specific issues (Manzano, 2014, p. 20)^3^. The creation of the UES in 1953 was part of this ongoing process.

Despite its short existence—less than 3 years –, the UES is one of the most famous Peronist institutions. Nevertheless, there is still no historical study entirely devoted to it. This historiographical gap is largely due to the lack of available sources, as many documents were lost or destroyed after the overthrow of the Peronist regime by a military coup in September 1955, the 18-years proscription of Peronism and the prosecution of its partisans. Fortunately, the press, Perón's speeches, the brochures and leaflets published by the propaganda services provided much information to the historians who tried to reconstitute the history of the UES (Page, 2011, p. 345–53; Rein R., 1998; Gambini, 2016). Another useful source is the reports on the UES activities drawn up by the commissions of investigation appointed by the ruling military junta, the so-called *Revolución Libertadora* (or “Liberating Revolution”), after the fall of Perón. These volumes reproduce original documents seized by the military junta's investigators within the UES and the Ministry of Education as well as the minutes of the interrogations conducted on members of the UES board. They provide many detailed information on the internal running of the institution, although, due to the context of production of these documents, they should be used and analyzed with caution and critical distance (Vice-Presidencia de la Nación, 1958, vol. I, p. 313–471; vol. II, p. 214–38).

Since its creation, the UES has been, and is still today, a controversial institution. Anti-Peronist pamphlets have perpetuated the memory of its scandalous aura, especially surrounding the women's section (Confalonieri, 1956; Rabinovitz, 1956; García, 1971). The installation of its headquarters in Perón's presidential residence shocked several sectors of the public opinion. The pictures of a widowed and aging Perón surrounded by young girls in casual sportswear reinforced the opponents' convictions about the corrupting and immoral nature of Peronism and fuelled suspicions about the President's private life. The case of Nelly Rivas, a young UES teenage girl who allegedly had a love affair with Perón and ended up living with him, was the culmination of this. As a symbol of the social and moral decadence produced by Peronism, the UES was for a long time the subject of a sulfurous reputation, forged and exploited from 1954 to 1955 onwards by the opposition, and especially by the Catholic Church, which was then in open conflict with the Peronist government (Caimari, 1994; Acha, 2011, p. 67–70).

It is only recently that several historians have tried to propose a different perspective on the UES, by situating it within the more general framework of youth and adolescent policies implemented by this political movement (Acha, 2011; Cammarota, 2014). This article intends to adopt a similar approach: reviewing the development of the UES shows how sport was conceived as a central element of the social and political Justicialist policies. It also contributes to a better understanding of the evolution of the regime's perception of youth and its role in society during the period. The UES is an original example of an attempt to provide State supervision of the leisure of a specific category: the secondary school and high school students. But it was also designed to include young people in the “*Nueva Argentina*” project developed by Perón by assigning them a proper place and mission within his political and social model of “Organized Community” (*Comunidad organizada*). This article aims to understand the objectives pursued by the Peronist government with the UES and to question the political dimension of this student institution.

## The UES: A Student Sports Organization

The exact conditions under which the UES was founded between 1952 and 1953 are still not well-known. The narrative put forward by the supporters of the military junta which deposed Perón has long prevailed. According to the reports of the commissions of investigation, the UES was set up by the Minister of Education to entertain Perón and to comfort him after the death of his wife, Eva Perón, in July 1952 (Vice-Presidencia de la Nación, 1958, p. 215–7). This new organization would therefore have been founded to “satisfy the vanity and personal pleasures” (Vice-Presidencia de la Nación, 1958, p. 217) of the president. Then, it would have been gradually erected as a “political instrument to achieve unconditional and total commitment of young people to the leader” (Vice-Presidencia de la Nación, 1958 p. 217). The UES would have been a key element of the “peronization” of youth. A more nuanced version proposed by researchers who have studied the history of the emergence of youth as a social and political category in Argentina is now opposed to it (Acha, 2011; Cammarota, 2014). Their analyses compare the creation of the UES with the measures taken by the Peronist government for young people and the regime's gradual recognition of this specific population category. The very name of the organization is significant in this respect: the Union of Secondary School Students. It highlighted the category of population for which the new institution was designed rather than its sporting and cultural dimension.

What does this name refer to? In Argentina as in many other countries, there are three levels of education: primary, secondary, and higher education (i.e., university). Secondary school students are therefore all those who have completed primary school and enter a secondary school: this can be a general high school (*colegio*), but also a specialized, technical or vocational school (Rein M. E., 1998). Under Peronism, the government sought to improve access to secondary education, especially for children from the working and middle classes (Cammarota, 2014, p. 36–45; Dussel and Pineau, 1995, p. 107–73). The Peronist leaders had several objectives: to enable more young people to continue their studies after primary school; to improve the qualifications of the workforce in general; and to open up access to university, in particular by abolishing the tuition fees (*arenceles*) that existed until 1951^4^. From that date, the public education system was entirely free of charge at all levels of education (Rein M. E., 1998, p. 42). All these measures were quite effective: the population of primary school leavers entering secondary school increased considerably (Manzano, 2014, p. 22). These guidelines of the Peronist educational policy set up frameworks to improve the social inclusion of youth. The creation of the UES in 1953 was a continuation of this policy: the government designed an organization specifically dedicated to secondary school students. The age of UES members was generally between 13 and 18, but could be as high as 25: the criterion for membership was not age, but the fact of being enrolled in any kind of secondary school.

Sport was the main and most visible activity offered to UES members, but it was not the only one. Adolescents could also engage in a variety of cultural and artistic practices, including drama, dance and singing^5^. However, the UES was supposed to be more than just a sports and cultural association. On November 16, 1953, at the inauguration of the headquarters of the men's section, Perón explained that “For some time now, the Ministry of Education has been thinking about the need to create organizations that bring together secondary school students, entrusting them with the administration and management of these organizations, so that young people can begin to manage and govern themselves at that age.” (Perón, 2001, p. 797). The motto of the UES magazine also defined it as a “student organization for students^6^”: it suggests that teenagers were supposed to run the organization themselves.

The UES was conceived as a space of sociability for high school students where they would learn collective responsibility and self-governance. As outlined by Perón in his inauguration speech, the primary purposes of the UES were to combat individualism, presented as one of the major issues of modern societies; to overcome differences of social origin and class; and to strengthen the sense of common belonging between members of the same generation (Perón, 2001 p. 797). In this sense, the UES was part of the political program of social transformation carried out by Peronist leaders.

The government aspired to set up sections throughout the country. However, this was still far from being a reality at the time of Perón's fall (Cammarota, 2010, p. 388). Outside Buenos Aires, the UES was based in several provincial capitals but it expanded very slowly: in many regions the UES was “an idea rather than a reality” (Acha, 2011, p. 71). In 1954, the magazine *Mundo Peronista* estimated that the women's section had a national membership of 60,000 and the men's 42,000, representing just over 50% of all secondary school students (Manzano, 2014, p. 24). These figures are significant, but they show that the UES was far from covering the entire secondary school population. In addition, official data were very likely to have been exaggerated by the government: membership did not always go hand in hand with active participation in the organization (Cammarota, 2014, p. 154–7).

Argentinean secondary school students did not have any obligation to join or participate in the activities of the UES. However, the authors of the 1958 investigation report stated that membership was neither free nor spontaneous and that an enrolment campaign was orchestrated from the Ministry of Education. The report states that the Buenos Aires secondary students were under constant pressure, even intimidation and threats (Vice-Presidencia de la Nación, 1958, vol. II, p. 217–8). Adrián Cammarota's research tends to refute the idea that UES delegates would have used coercive methods to enroll their peers. According to the testimony of a former UES female leader, their role was to distribute membership cards during high school sports competitions. And they were not always successful: in Cammarota's detailed case study, the Morón public high school, only a minority of students joined the UES (Cammarota, 2014, p. 155).

## A Model Organization and A Showcase for the Regime

To recruit new members, the government did not necessarily need to coerce teenagers. The success of the UES can be explained by its many attractions. The Peronist leaders invested massively to develop the new organization, as evidenced by the exceptional facilities enjoyed by the UES affiliates. In accordance with the social standards of the time, the organization was divided into two sections: teenage girls and boys carried out their activities separately, with a few exceptions (Unión de Estudiantes Secundarios, 1954)^7^. Otherwise, each section had its own headquarters, originally located in the most elegant downtown areas of Buenos Aires. Soon, they were also provided with large sports fields and improved facilities: the girls' section was established in the presidential residence, while the male adolescents settled in the Nuñez neighborhood in the north of the capital^8^. This venue comprised a total of six hectares, including several fields previously occupied by different clubs. Some of these clubs had been dislodged by the City Council so that the Peronist authorities could recover the land—as well as the facilities that had been built on it—and transfer it to the UES (Hémeury, 2018, p. 197 and 329–38).

These decisions provoked strong protests from the leaders of the expropriated clubs. They were also heavily criticized by political opponents who denounced the “inappropriate luxury” (Vice-Presidencia de la Nación, 1958, vol. II, p. 215) of the UES facilities. They were indeed outstanding. The men's section activity center included an indoor swimming pool, a movie theater, a dining hall, dormitories, kitchens, and gymnasiums, 18 outdoor basketball courts, nine football fields, and 11 tennis courts. Several pavilions were used to house the administrative services, the infirmary, and the “American bar” with its billiard tables, a garage and a repair workshop for the members' self-service scooters (*motonetas*) (Vice-Presidencia de la Nación, 1958, vol. I, p. 362). The women's section enjoyed similar amenities. The UES girls could practice fencing, gymnastics, basketball, volleyball, athletics, or canoeing and ride *motonetas*. The women's section alone employed a total of 320 people (Vice-Presidencia de la Nación, 1958, p. 392).

The government turned the UES into an unprecedented model institution. It quickly occupied an important role in the regime's propaganda, showing the government's action in favor of the development of sports practice and its physical and educative benefits for the youth^9^. The UES was also closely associated with official public events. Its members took part in the regime's celebrations, parading in the streets and singing their own march, which was an ode to the president and to the “New Argentina^10^.” In addition, Perón often visited the UES facilities: he attended lessons, sometimes taking part in them, and handed out prizes and awards to the winners of the competitions (Cammarota, 2014, p. 155). This is why anti-Peronists and the *Revolución Libertadora* identified the UES as an avatar of the Fascist and Nazi youth movements. For them, without a doubt, it was a political organization, akin to a juvenile wing of the Peronist Party, designed by the highest leaders of the Peronist movement and controlled from the top of the State. A review of the internal running of the institution seems to prove them right.

## A Parastatal Organization

In theory, the UES adolescents were supposed to learn how to self-organize and collectively manage an institution presented as their own. This narrative referred to the principles of the Peronist Doctrine which valued team-spirit, general interest and collective commitment^11^. The UES emerged as a means of integrating a specific group (i.e., young people) into the “Organized Community” project forged by Peronism. Perón advocated a corporatist and top-down conception of society. In this system, each sector of the society should run its own organizations and the State would act as an impartial arbiter, guaranteeing social harmony (Acha, 2011, p. 57–8)^12^. But, in the UES as in many other Peronist organizations, this learning of autonomy and self-governance took place under the patronage of the State. Although the members of the organization's board were young students, the institution was in fact operated by employees of the Ministry of Education. After the overthrow of the regime, former leaders of the UES were interrogated by the commission of investigation. They acknowledged that they all had connections with the Ministry and that their functions were, most of the time, “purely formal” (Vice-Presidencia de la Nación, 1958, vol. II, p. 218). Méndez San Martín and his staff took all the fundamental decisions regarding the institution. The funding allocated to the UES came from the Ministry of Education's budget: according to the investigators, over 270 million pesos had been granted to the youth organization in 3 years (Vice-Presidencia de la Nación, 1958, vol. I, p. 314–6).

The UES, undoubtedly, was a parastatal organism: theoretically, it was an independent institution, but in practice, it was closely tied to the regime. Still, the creation of this institution shows the shift in Peronist leaders' attitudes toward youth. The social and political context of the early 1950's in Argentina partly explains this change. From 1952–1953 onwards, the Peronist government sought to ensure the continuity of its increasingly contested political project by relying on teenagers. These “citizens in the making” needed to be trained and prepared to continue the work of General Perón.

With the UES, the government intended to address a category of the population which until that date had not been the target of specific political measures. In his speeches addressed to the UES members, Perón gave the teenagers a place in the “*Nueva Argentina*,” by assigning them a mission, and a role to fulfill (Perón, 2001, p. 82). These were both patriotic and political: young people had to take part in national development. Their experience at the UES would enable them to acquire a sense of responsibility and to become enlightened citizens who could perpetuate the task undertaken by Peronism. For Peronist leaders, the institution had to be concurrently a place for the entertainment of young people and a place for socialization and civic education to raise political awareness. And for them, the ideal citizen was necessarily a Peronist citizen.

But the impact of this youth mobilization endeavor was in fact limited. Even the authors of the UES investigation report noted that many adolescents would have “come only to obtain some benefit from this inexhaustible cornucopia” (Vice-Presidencia de la Nación, 1958, vol. II, p. 226). This remark contradicts the idea that the UES would have successfully “peronized” its members on a long-term basis. Indeed, many young people would have joined the organization only because they were attracted by the significant bonuses, material rewards, and opportunities offered by the institution. During the sports tournaments, participants could win a motorbike, money, or jewelry. According to the UES investigation report, some of them could even be rewarded with a position in the public administration or housing for those who requested it (Vice-Presidencia de la Nación, 1958).

O. Acha and A. Cammarota's studies also underline the mixed success of the UES and the low impact it had on the adolescents of the period. Their analyses invite a cautious examination of the triumphalist declarations of Peronist leaders as well as the exaggerations of the supporters of the *Revolución Libertadora*. Both researchers stress the late and somewhat improvised nature of the Peronist political program for youth. If the UES indeed “constituted a massive project of youth organization, Peronist indoctrination and the construction of a new generation” (Acha, 2011, p. 71), its members were never really trained and prepared for political activism. This became very clear during the long conflict between the Argentinean Catholic Church and the regime: the UES was unable to compete with the militant Catholic youth organizations (Acha, 2011, p. 72–3). Young people never gained any real autonomy as political actors, “capable of concretely acting on reality.” (Acha, 2011, p. 77) The government's attitude toward youth was marked by a paternalistic approach, as reflected in the management of the UES. Despite the promises of the President, the teenagers were always in a subordinate position.

## Conclusion: Contradictions and Limits of the UES

The creation of the UES was a political gesture: as a space for socialization, the new organization would contribute to the governmental project of social transformation. Although the UES was strongly marked by a partisan and propagandistic dimension, it was not, strictly speaking, a political organization. The organization never became the “Perón Youth” or the juvenile section of the Peronist Party, maybe because the recognition of teenagers as a specific category came late and remained unfinished. The UES project itself was contradictory, since the autonomy promised and announced to the members turned out to be extremely limited.

The UES was a relatively short experience that failed to make a significant impact on adolescents, at least politically. However, it confirmed the central place given to sport by the government to reach young people (Gagliano, 1995). The institution was used by the regime to showcase an idealized representation of youth and the Peronist “happy world” (Gené, 2008): a cheerful, athletic, virtuous youth that would gradually emancipate itself, under the benevolent protection of Perón. But the “peronization” of young people seems to have been superficial: the UES was not a space for political struggle during the 1955 military coup. Nor does it seem to have been the source of clandestine networks of Peronist resistance during the dictatorship of the *Revolución Libertadora*. However, it was considered one of the outstanding institutions of early Peronism, as shown by the re-creation of an UES by revolutionary Peronist groups in the early 1970's. As for many creations of Peronism, the history of the UES did not end with its dissolution.

## Data Availability Statement

The original contributions presented in the study are included in the article/supplementary material, further inquiries can be directed to the corresponding author/s.

## Author Contributions

The author confirms being the sole contributor of this work and has approved it for publication. All quotes have been translated from Spanish into English by LH.

## Conflict of Interest

The author declares that the research was conducted in the absence of any commercial or financial relationships that could be construed as a potential conflict of interest.

## Footnotes

^1^Perón (1949), *La gimnasia y los deportes*, p. 5.

^2^There are many references in the sources to this Peronist motto. See, for example, Servicio Internacional de Publicaciones Argentinas (SIPA), *Infancia privilegiada*, Buenos Aires. p. 49.

^3^In her study of the Argentine youth culture between the 1950s and the 1970s, Valeria Manzano proposes, as the title of her first chapter, a pertinent expression to designate the transformations at work under Peronism: “Carving Out a Place for Youth.”

^4^This measure was announced by Perón as early as 1949. On free public education, see Servicio Internacional de Publicaciones Argentinas (SIPA), *S*í*ntesis de la Educación en la Argentina*, Buenos Aires. p. 54.

^5^See the magazine *UES*, Año I, n°4, September 1954 and (Unión de Estudiantes Secundarios, 1954). *Teatro Argentino*.

^6^Front page of the magazine of *UES*, Año I, no. 4, september 1954: “*de los estudiantes para los estudiantes*.”

^7^Teenagers of both sexes were often together during fencing demonstrations and shared the same yacht club. The UES section in the province of Eva Perón (La Pampa) put on a play that brought boys and girls together on stage.

^8^The men's section moved to No. 1050 Republiquetas Street, currently Miguel B. Sánchez Street. The facilities still exist and now belongs to the Centro Nacional de Alto Rendimiento Deportivo (CeNARD), run by the Argentinean State Department for Sports.

^9^The press of the period, and in particular the newspapers controlled by the Peronist government, such as *Mundo Peronista*, reported extensively on the activities of the UES. For a more detailed study of the UES media coverage, see (Sénen Gónzalez and Bosoer, 2019).

^10^The UES march's lyrics are published in Unión de Estudiantes Secundarios (1954). *Teatro Argentino*.

^11^Many official publications laid out the Peronist doctrine and its principles: for example, Granata (1954), p. 15–8.

^12^O. Acha stresses the influence of the social-Catholic movement in Perón's doctrine. The Peronists recognized the importance of the society's “intermediate bodies” as the only guarantee of social harmony.

## References

[B1] Acha O. (2011). Los muchachos peronistas. Orígenes olvidados de la Juventud Peronista (1945–1955). Buenos Aires: Planeta.

[B2] Barry C. Ramacciotti K. Valobra A. (2008). La Fundación Eva Perón y las mujeres: entre la provocación y la inclusión. Buenos Aires: Biblos.

[B3] Caimari L. (1994). Perón y la Iglesia Católica. Religión, Estado y sociedad en la Argentina (1943–1955). Buenos Aires: Ariel.

[B4] Cammarota A. (2010). Una juventud responsable, disciplinada y peronista. La *Revista de la Unión de Estudiantes Secundarios* (1954-1955), in Ideas y debates para la Nueva Argentina. Revistas culturales y políticas del peronismo (1946-1955) Vol. II, eds. C. Panella and G. Korn. (La Plata: Universidad Nacional de La Plata), 387–405.

[B5] Cammarota A. (2014). Somos Bachiyeres. Juventud, cultura escolar y peronismo en el colegio nacional mixto de morón (1949–1969). Buenos Aires: Biblos.

[B6] Confalonieri O. D. (1956). Perón contra perón. Buenos Aires: Antygua.

[B7] Darbon S. (2008). Diffusion des sports et impérialisme anglo-saxon. De l'histoire événementielle à l'anthropologie. Paris: Éditions de la Maison des Sciences de l'Homme. 10.4000/books.editionsmsh.1035

[B8] Dussel I. Pineau P. (1995). De cuando la clase obrera entró al paraíso: la educación técnica estatal en el primer peronismo, in Historia de la educación en la Argentina. Vol. VI, ed A. Puiggrós (Buenos Aires: Galerna), 107–73.

[B9] Gagliano R. (1995). Consideraciones sobre la adolescencia en el período, in Historia de la educación en la Argentina. Vol. VI, ed A. Puiggrós (Buenos Aires: Galerna), 170–203.

[B10] Gambini H. (2016). Historia del peronismo. Vol. 2. La obsecuencia (1951–1955). Buenos Aires: Ediciones B Argentina.

[B11] García E. A. (1971). Yo fui testigo. Antes, durante y después de la segunda tiranía. Memorias. Buenos Aires: Laserre.

[B12] Gené M. (2008). Un mundo feliz. Imágenes de los trabajadores en el primer peronismo, 1946–1955. Buenos Aires: Fondo de Cultura Económica.

[B13] Granata M. (1954). Pueblo y peronismo. Buenos Aires: Presidencia de la Nación – Secretaría de Prensa y Difusión.

[B14] Hémeury L. (2018). Le pacte introuvable. Sport, peronisme et societe en Argentine (1946–1955). (Ph.D. thesis). Sorbonne-Nouvelle Paris 3 University, Paris.

[B15] Lanfranchi P. (2000). Entre initiative privée et question nationale. Genèse et évolution des politiques sportives en Europe (Grande-Bretagne, Allemagne, France et Italie). Politix. 13:50 10.3406/polix.2000.1085

[B16] Manzano V. (2014). The Age of Youth. Culture, Politics and Sexuality from Perón to Videla. Chapel Hill: University of North Carolina.

[B17] Page J. A. (2011). Perón. Una biografía. Buenos Aires: Delbolsillo.

[B18] Perón J. D. (1949). La gimnasia y los deportes.

[B19] Perón J. D. (2001). Obras completas. Buenos Aires: Fundación pro Universidad de la Producción y del Trabajo.

[B20] Plotkin M. B. (2003). Mañana es San Perón. A Cultural History of Perón's Argentina. Wilmington: SR Books.

[B21] Rabinovitz B. (1956). Sucedió en la Argentina (1943–1956). Lo que no se dijo. Buenos Aires: Gure.

[B22] Rein M. E. (1998). Politics and Education in Argentina 1946–1962. New York, NY: M. E. Sharpe.

[B23] Rein R. (1998). ‘El Primer Deportista': the Political Use and Abuse of Sport in Peronist Argentina. Int. J. Hist. Sport. 15, 54–76. 10.1080/09523369808714028

[B24] Sénen Gónzalez S. Bosoer F. (2019) Perón, juventud y deporte: la experiencia de la Unión de Estudiantes Secundarios, in El deporte en el primer peronismo, eds R. Rein R. C. Panella (La Plata: EPC), 124–46.

[B25] Unión de Estudiantes Secundarios (1954). Teatro Argentino. Función Extraordinaria, En Homenaje al Excelentísimo Señor Presidente de la Nación Argentina, Juan D. Perón y a su Excelencia el Señor Gobernador de la Provincia de Buenos Aires Don Carlos Aloé, organizada por la U.E.S. Eva Perón 30 de julio de 1954. Buenos Aires.

[B26] Vice-Presidencia de la Nación (1958). Documentación, autores y cómplices de la irregularidades cometidas durante la Segunda Tiranía. Buenos Aires.

